# Effect of surface charge on wettability and electrolyte behavior on graphene surfaces using molecular dynamic simulation

**DOI:** 10.1038/s41598-025-02331-1

**Published:** 2025-05-20

**Authors:** Mukesh Kumar, Santosh Kumar Tamang, Maryom Dabi, Anil Kumar, Ankur Jaiswal

**Affiliations:** 1 Department of Mechanical Engineering, North Eastern Regional Institute of Science and Technology, Nirjuli, Arunachal Pradesh 791109 India; 2 Department of Mechanical Engineering, Bhilai Institute of Technology, Durg, Chhattisgarh India; 3 Department of Mechatronics, Manipal Institute of Technology, Manipal Academy of Higher Education (MAHE), Manipal, Karnataka 576104 India

**Keywords:** Graphene, Surface charge, Wettability, Molecular dynamics simulations, Electrolyte behavior, Electrostatic interactions, Materials science, Mechanical engineering

## Abstract

The interaction between electrolytes and graphene surfaces is critical in applications such as electro-wetting and energy storage. This study employs molecular dynamics simulations to investigate the influence of surface charge on electrolyte wettability and behavior. At 0.00 eV, the contact angle is 30.33°, indicating high wettability, while an increase in surface charge reduces wettability, with the contact angle rising to 36.88° at 0.06 eV and stabilizing at 62.30° at 0.15 eV. The lateral droplet spread decreases from 37.56 nm to 34.78 nm, indicating a more compact electrolyte distribution. Temperature simulations reveal a sharp rise exceeding 2000 K within picoseconds, peaking at 2800 K at 0.15 eV before stabilizing between 100 and 200 K. Potential energy increases from 0.081 $\:\times\:$ 10^7^ kcal/mol to 3.520 $\:\times\:$ 10^7^ kcal/mol, reflecting stronger electrostatic interactions. Additionally, the electric force rises with charge, consistent with Coulomb’s law, while the diffusion coefficient decreases from 8.0 Å^2^/ps to 6.0 Å^2^/ps, indicating reduced particle mobility. These findings enhance the understanding of surface charge effects on electrolyte behavior, contributing to the optimization of electro-wetting and energy storage applications.

## Introduction

Graphene is a single layer of carbon atoms that have numerous excellent mechanical, electrical, thermal, optical, and chemical characteristics. Application includes electronics (high speed transistors flexible electronics), energy storage (batteries, supercapacitors), composites (light-weight material), biomedical (drug delivery, biosensors). Other applications of graphene include filtration using water, catalysis and sensing. However, it has its caveat of scale, cost of production and impacts on the environment etc. The present study focuses on energy storage systems, where wettability plays a critical role in defining the surface response at the nanoscale in devices such as supercapacitors, batteries, and fuel cells. There is an urgent need to directly control the nucleation kinetics in these systems to enhance their performance and efficiency. Molecular dynamics simulations (MDS) have proven to be valuable tools for wettability analysis, as they offer insights into the atomic-scale structural, energetic, and temporal characteristics of a graphene-electrolyte interface. Song et al.^1^ investigates the transformative effects of electric fields on the morphology and dynamics of polar water droplets. The study demonstrated that weak electric fields cause asymmetric droplet spreading, with a disparity in leading and trailing contact angles, while stronger fields induce symmetric spreading by reorienting water molecules and altering hydrogen bonding networks. Their findings underline the intricate interplay between electric fields, water-water interactions, and water-solid forces in dictating droplet behavior on surfaces. Song et al.^2^ extended these insights to nanoscale droplets on polar silica substrates, identifying dynamic wetting stages under electric fields up to 1.0 V/nm. The research highlighted the role of molecular orientation and hydrogen bond modification in shaping droplet asymmetry and spreading behavior. Similarly, Nikzad et al.^3^ explored how parallel and perpendicular electric fields influence the water liquid-vapor interface, noting changes in density distribution, hydrogen bonding, and surface tension, further elucidating the molecular-scale interactions governing droplet behavior under electric fields. Song et al.^4^ the reported ionic liquid droplets under electric fields, revealing layered ionic distributions and asymmetric wetting. Electric fields reduced static contact angles while promoting asymmetry through differential ion diffusion, particularly on hydrophilic surfaces. This interplay between electric forces, ionic interactions, and substrate properties exemplifies the complexity of electrowetting phenomena. Sofos et al.^5^ demonstrated the practical application of electric fields in nanochannel ion removal, where field strength dictated ion transport dynamics and equilibrium times. These findings parallel the work of Jiang et al.^6^ studied electric double layer (EDL) formation in graphene-based capacitors, emphasizing ion-specific structuring and its impact on energy storage efficiency. Graphene’s unique wetting characteristics were extensively explored by multiple studies. Andrews et al.^7^ noted that water droplets form distinct patches on graphene, influenced by substrate hydrophilicity. Meanwhile, Lai et al.^8^ investigated the evolution of graphene’s wettability, transitioning from hydrophilic to hydrophobic due to ambient adsorption. Wei et al.^9^ investigated graphene/electrolyte interfaces, showing asymmetric interfacial tension responses under potential bias, which influenced wettability without altering EDL formation. Similarly, Wang et al.^10^ observed that graphene’s wettability is modulated by charge density, transitioning from hydrophobic to superhydrophilic under certain conditions. Graphene’s functionality extends to energy storage and recovery systems. Shim et al.^11^ found that ionic liquids enhance the specific capacitance of graphene supercapacitors, while Bi et al.^12^ connected molecular dynamics data to macro-scale performance, predicting capacitance in MOF-based supercapacitors. Vatamanu et al.^13^ emphasized the role of nanostructured carbon electrodes in enhancing energy density and charge dynamics in supercapacitors. Huang et al.^14^ and Zhao et al.^15^ provided comprehensive reviews on graphene’s intrinsic wettability and its implications for electrochemical devices. They highlighted ongoing challenges in optimizing graphene synthesis and wettability control to improve its adoption in energy-related fields. Defects and external factors significantly influence graphene’s wetting behavior. Xao et al.^16^ emphasized the role of defects in graphene coatings, leading to high contact angle hysteresis. In contrast, Yusuff et al.^17^ demonstrated graphene’s effectiveness in reducing oil viscosity, showcasing its versatility in enhancing fluid dynamics. Sophisticated MD simulations have provided nuanced insights into wetting and interfacial behaviors. Pykal et al.^18^ employed polarization models to study electrolyte structuring at graphene interfaces, while Hung et al.^19^ utilized free energy calculations to explore adhesion mechanisms on graphene-coated surfaces. These methodological advancements are pivotal for unraveling the complexities of molecular interactions and guiding material design for energy and wetting applications. Poorsargol et al.^20^ employs molecular dynamics simulations to study the adsorption of mixed cationic and anionic surfactants on armchair graphene, exploring the effects of temperature, electrolyte, and alcohol in the aqueous solution. The results demonstrate that adding an electrolyte enhances system stability by screening electrostatic repulsion between surfactant head groups, leading to denser surfactant accumulation on the graphene surface. However, increasing temperature reduces stability by promoting surfactant desorption. The absence of alcohol strengthens interactions between surfactants and graphene, further improving system stability. Olabi et al.^21^ explores the use of graphene in energy storage devices, absorbers, and electrochemical sensors. The study highlights the need to address the limitations of graphene to enhance its performance. It emphasizes the expanding applications of graphene in electroanalytical and electrochemical sensors and suggests that advancements in graphene synthesis and a deeper molecular understanding of graphene oxide will improve its applicability. The wettability of graphene with EMIMBF_4_ electrolyte is crucial for optimizing energy storage systems like supercapacitors and batteries. It ensures efficient ion transport and uniform electrolyte distribution across the graphene surface, enhancing performance, charge-discharge cycles, and stability. MDS are essential for understanding the atomic-scale interactions between the electrolyte and graphene. They help optimize wettability by studying surface modifications and guide the design of efficient materials with tailored properties for improved performance. MD simulations reduce experimental costs and time by predicting material behavior, accelerating the development of advanced energy storage systems with better efficiency and longevity.

To address the literature gap, this work focuses on studying the wettability of graphene under varying surface charge conditions at a constant electric field (1.0 V/nm) applied along the Z-direction through MDS. Further, the present work investigates the surface wettability of graphene for the application of energy storage system considering electric force, EMIMBF_4_ electrolyte, resistance forces, density, mean square displacement (MSD), diffusion coefficient, electrolyte temperature, energy. The simulations were carried out in Large atomic molecular massive simulator (LAMMPS) and results of these simulations were analyzed and visualized using scientific software tools such as OVITO and VMD.

## Materials and methods

### EMIMBF_4_ electrolyte

EMIMBF_4_ also known as 1-ethyl-3-methylimidazolium tetrafluoroborate, [C_6_H_11_N_2_][BF_4_] is an ionic liquid having a potential use in energy storage area. Due to its high ionic conductivity and rather wide electrochemical stability range (4–6 V), the compound is suitable for use in supercapacitors and lithium-ion batteries. They also assured of high thermal and chemical stability of EMIMBF_4_ making it suitable to work in high temperatures. Compared with traditional electrolytes, it provides safety profiles of non-volatility and non-flammability. Composable carbon-based materials such as graphene have beneficial features in charge transport and solid-electrolyte interphase (SEI) stability. It also develops an environmental advantage as a green electrolyte for EMIMBF_4_. These features make it more suitable for new generation batteries, encouraging passiveness in power storages of present day. Therefore, in the present study, the EMIMBF_4_ electrolyte has been chosen to investigate graphene surface wettability.

### Computational modeling and simulation setup

**Fig. 1:**
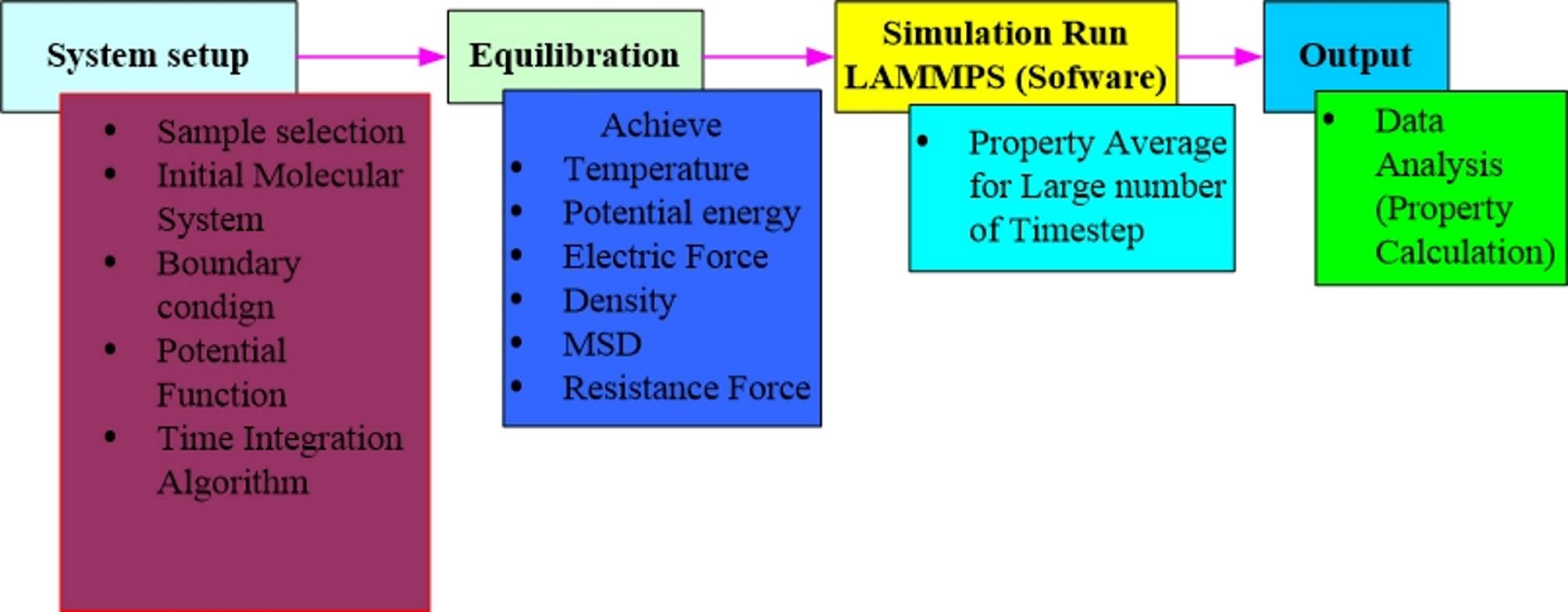
General description of MD simulation method applied to simulate vaporizing of water phenomena.

**Fig. 2:**
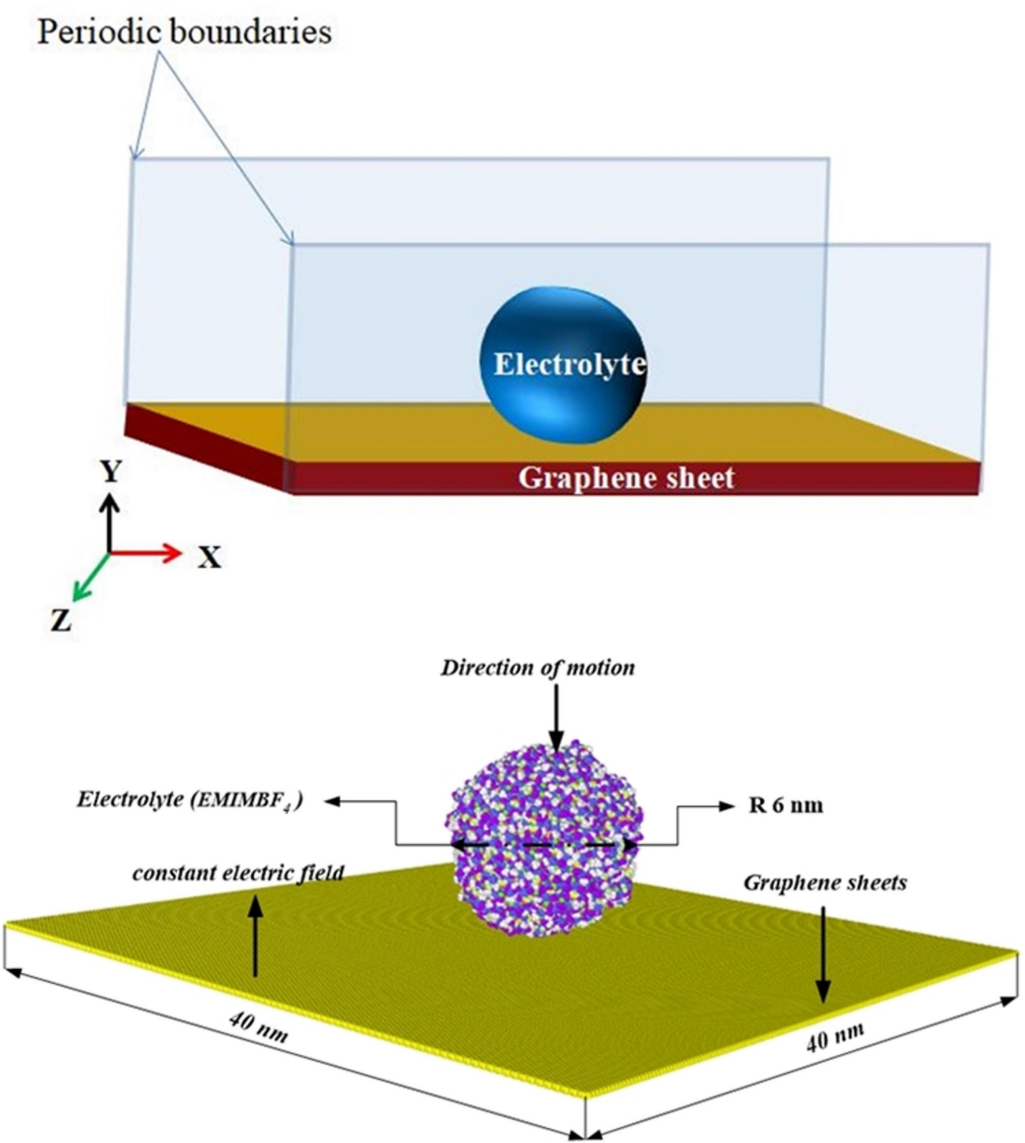
(**a**) A schematic representation of the EMIMBF_4_ electrolyte nanodrop simulation on the graphene surface is shown. (**b**) A screenshot of the initial atomic configuration and the dimensions of graphene surface visualize EMIMBF_4_ electrolyte molecules using the OVITO software before activation, a perspective view of the interatomic potential.

Figure 1 shows the steps involved in MDS simulation using LAMMPS. All molecular dynamics simulations have been carried out using the LAMMPS (Large-scale atomic/molecular massively parallel simulator) package software. The simulated system has a graphene substrate for the electrode and an EMIMBF_4_ electrolyte nanodroplet. The size distributions for the initial droplet are centered 0 Å from the substrate surface in the z direction, which was obtained using Eq. (1). In this work, to model the initial stage of the interaction of the electrolyte nanodroplet with the graphene surface, geometries with sizes 400 $\:\times\:$ 400 $\:\times\:$ 1 (Å)^3^ and the size of nanodroplet is 120 Å diameter are used in this study. To have sufficient space for the nanodroplet to spread and to bounce completely, periodic barriers were introduced on the simulated system in all the three domains. These boundaries were characterized by dimensions of 400 Å in the x and y planes and 300 Å in the Z plane. The hexagonal array with a repeated distance of 3.46 Å comprises graphene atoms as the flat base of the structure are shown in Fig. 2.

The force field forms for each molecule in the domain are now solved using molecular dynamics using Eq. (1) for every interval during the simulation run.

1 $$\:{\:F}_{i\:}=\sum\:_{j\ne\:i}^{N}-{\nabla\:E}_{ij}\left({r}_{ij}\right)$$

*N*, ${F_i}$ and ${r_i}$ are the total number of molecules, force and position respectively. To compute short-range interactions, Lennard–Jones (LJ) and Coulomb electrostatic potential functions are combined^22^. The modified Lennard–Jones (LJ) approach for modeling interaction energies in electrolytes provides a simplified framework to capture pairwise interactions, incorporating both repulsive and attractive forces. However, its adequacy in describing all peculiarities of electrolyte systems is limited. The LJ potential primarily accounts for Vander Waals interactions and is typically parameterized to reproduce bulk properties such as density or radial distribution functions. While effective for isotropic interactions, it often fails to fully capture the complex, anisotropic interactions inherent in electrolytes, such as ion-solvent or ion-ion interactions influenced by charge distributions, solvation shells, and dielectric screening effects. These require additional terms, such as Coulombic interactions or polarizable force fields, to achieve a more comprehensive description^23^.

According to dihedral angles and angular conformations, the modified LJ approach is generally inadequate^24^. Dihedral angles, which govern the torsional flexibility of molecular systems, and angular conformations, which dictate bond angle preferences, are poorly described by pairwise LJ potentials alone^25^. These structural features depend on intramolecular interactions that require explicit bonded terms (e.g., harmonic potentials for bond angles or cosine-based potentials for dihedrals) commonly found in molecular force fields like CHARMM. In electrolyte systems, where ion coordination and solvent molecule orientations play critical roles, the lack of such terms in a modified LJ approach can lead to inaccurate conformational sampling and unrealistic structural dynamics. To address these limitations, hybrid models combining the modified LJ potential with electrostatic and bonded interaction terms are recommended. Such models can better reproduce the structural and dynamic peculiarities of electrolytes, including proper dihedral and angular distributions^26^. This work assumes that the modified LJ potential (${E_{ij}}$) offers the property between different types of atoms of a soft repulsive core, which is adjustable by an activation parameter$\lambda$^27^. In order to prevent singularities during free energy calculations when sites are produced or destroyed, these pair styles include a soft repulsive core that is controllable via the parameter $\lambda$ The pair interaction disappears with a soft repulsive core when $\lambda$ approaches to 0. The pair interaction approaches the typical, non-soft potential when lambda trends towards 1. Beutler et al.^28^ provides the interatomic potential for the modified LJ, as given in Eq. (2), in functional form.

2 $$\:{E}_{ij}=\:{\lambda\:}^{n}4{\epsilon\:}_{ij}\left\{\left(\frac{1}{{\left[{\alpha\:}_{LJ}{\left(1-\lambda\:\right)}^{2}+{\left(\frac{r}{\sigma\:}\right)}^{6}\right]}^{2}}\right)-\left(\frac{1}{\left[{\alpha\:}_{LJ}{\left(1-\lambda\:\right)}^{2}+{\left(\frac{r}{\sigma\:}\right)}^{6}\right]}\right)\right\}+{\lambda\:}^{n}\frac{{q}_{i}{q}_{j}}{4\pi\:{\epsilon\:}_{0}{\epsilon\:}_{r}\left[{\alpha\:}_{c}{\left(1-\lambda\:\right)}^{2}+{r}^{2}\right]}$$

where $\:{E}_{ij}$ is the interatomic potential between them $\:{i}^{th}$and $\:{j}^{th}$ atoms of a molecule, $\:\sigma\:\:$is the distance at which the inter atomic potential is zero, $\:r$ is the separation between them, $\:{\varvec{\epsilon\:}}_{0}{\varvec{\epsilon\:}}_{\varvec{r}\:}$ is the medium’s dielectric constant. $\:{\alpha\:}_{LJ}$is soft core LJ coefficient $\:{\alpha\:}_{C}\:$is coulomb coefficient, $\:q\:$is the change on atomic site, $\:n\:$is scaling factor, $\:{\epsilon\:}_{ij\:}$is the depth of potential well, $\:\lambda\:\:\:$is the activation parameters. The values for the coefficients $\:{\alpha\:}_{LJ}$ and $\:{\alpha\:}_{C}$are 0.5Å and 10Å, respectively In order to initially generate a hydrophobic bond between water molecules and solid platinum substrate, the activation parameter is set to $\lambda$ = 0.95, and it is set to $\lambda$ = 1 for other atomic contact circumstances.

The Lorentz–Berthelot mixing principles are used to determine the cross-atomic interactions between graphene and electrolyte atoms in this case, as shown in Eqs. (3, 4)

3 $$\:{\sigma\:}_{mn}=\:\frac{{\sigma\:}_{m}+{\sigma\:}_{n}}{2}$$

4 $$\:{\epsilon\:}_{mn}=\:\sqrt{{\epsilon\:}_{m}}\times\:\sqrt{{\epsilon\:}_{n}}$$

where $\:{\:\sigma\:}_{m}$, $\:{\epsilon\:}_{m}$, $\:{\sigma\:}_{n}$ and $\:{\epsilon\:}_{n}$ are parameters of 12−6 LJ potential for atom m and n; $\:{\sigma\:}_{mn}$and $\:{\epsilon\:}_{mn}$ are parameters of 12−6 potential between atoms m and n. In Table 1 show the interaction parameters for nitrogen, carbon, hydrogen, boron, fluorine, graphene. It is with the help of L-J (12−6) potentials that the dynamics of the interactions between Graphene atoms and the electrolyte molecules are modeled. Table 1 presented the potential parameters also in this study. As a result, the embedded atom model potential can be used to model the behavior of graphene atoms and their interactivity. Long-range electrostatic interactions are computed using the Particle-Particle Particle-Mesh (PPPM) algorithm with 10^− 6^ tolerance.

**Table 1:** Interaction parameters of EMIMBF_4_ electrolyte molecules and graphene surface.

Atom type	$\:{{\epsilon\:}}_{{m}{n}}$ (kcal/mole)	$\:{{\sigma\:}}_{{m}{n}}$ (Å)	m_a_ (u)
N-N	0.20	3.50	14.007
C-C	0.30	3.40	12.011
H-H	0.10	2.50	1.008
B-B	0.15	3.00	10.811
F-F	0.25	3.0	18.998
Graphine–Graphine	0.30	3.40	12.011

**Fig. 3:**
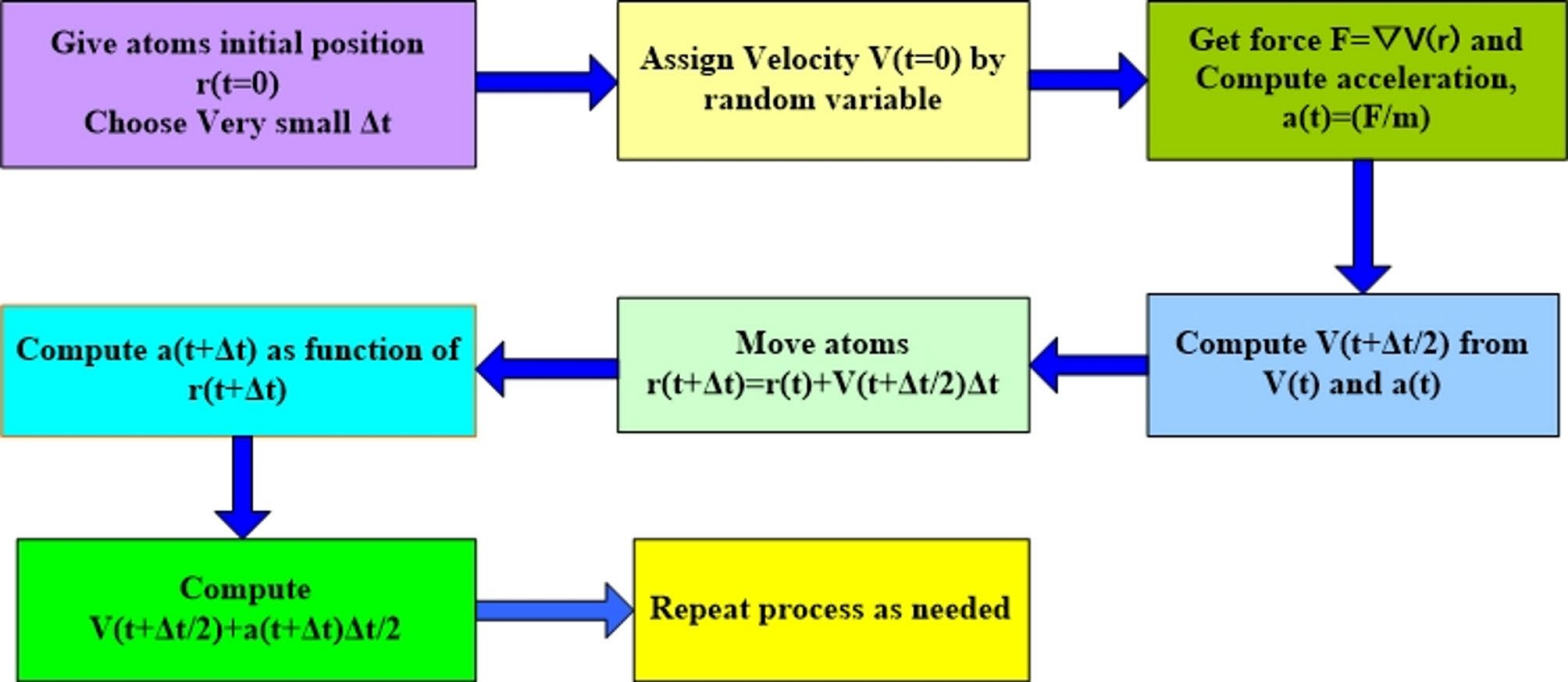
Flowchart showing the implementation of the velocity-Verlet algorithm.

For both the short range and the long range forces the spherically truncated methods are applied with the truncation distances of 10 Å. The SHAKE algorithm is applied to maintain its binding length and angle of the electrolyte molecule within all simulations. The motion for all particles is described with the help of the Velocity-Verlet algorithm with a time step of 1 fs on average. The algorithm for equilibration of the process performance variables is implemented through more intricate procedures and mathematical calculations, as seen in Fig. 3. Using the Nosé-Hoover thermostat, the system is equilibrated at a temperature of 300 K in the NVT ensemble to achieve thermal equilibrium. The nanodroplet is placed on the surface, and the system is then simulated using the NVE ensemble. The resulting atomic trajectories can be visualized using OVITO software.

## Results and discussion

**Fig. 4:**
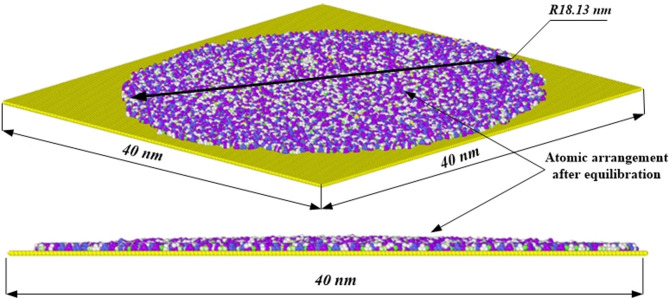
Perspective and frontal plan views of the atomic positions of the EMIMBF_4_ electrolyte molecules adsorbed at the graphene surface after interaction at 100 ps in the zero-charge condition.

Figure 4 depicts the snapshot of an electrolyte droplet which has been placed over a graphene substrate surface and observed at 100 ps of molecular dynamics simulation. The top view demonstrates the structure of the electrolyte layer when it has reached the intended radial dimension of the anode over the 40 nm $\:\times\:$ 40 nm graphene surface, while the side view is used to depict the thickness of the layer and its homogeneity. Looking at the top view, the electrolyte has an almost circular shape and has a diameter of around 36.26 nm thus establishing it extended of the graphene substrate. The “atomic arrangement after equilibration” label also points towards the fact that this structure constitutes a stable state of the electrolyte on substrate once the simulation has been run for sufficiently long time. The side view also displays the layering effect as well as the way the electrolyte adheres to the graphene structure together with its distribution over a width of 40 nm. This consistent layering also demonstrates that the structure is stable, and the effect of the electrolyte molecules on the graphene substrate is stable as well. This equilibrium structure far illustrates the ability of the electrolyte to form a stable, layer-like array where the molecules are spaced out on the graphene substrate, and this is measured by the forces within the molecules of the electrolyte or between the electrolyte and the substrate^29^. This kind of insight enriches knowledge on the nature of interaction with electrolytes in systems containing graphene and similar conductive materials, which are highly important for the development of new energy storage and nanofluidic technologies.

### Simulation parameters

The simulation parameters including the electric field in Z direction and the surface charge at graphene surface are shown in Table 2 based on previous work^6,30^. The table also shows that a constant electric field of 1.0 V/Å is applied in the Z-direction of the graphene surface, and the charge of the graphene surface is varied by 0.03 eV. Such systematic variation has been planned to study the effect of surface charge for the electrolyte solutions according to the predetermined parameters as mentioned earlier.

**Table 2:** Different parameters of applied on graphene surface.

cases	Electric field along Z direction (V/Å)	Applied surface charge (eV)
1	1.0	0.00
2	1.0	0.03
3	1.0	0.06
4	1.0	0.09
5	1.0	0.12
6	1.0	0.15

**Fig. 5:**
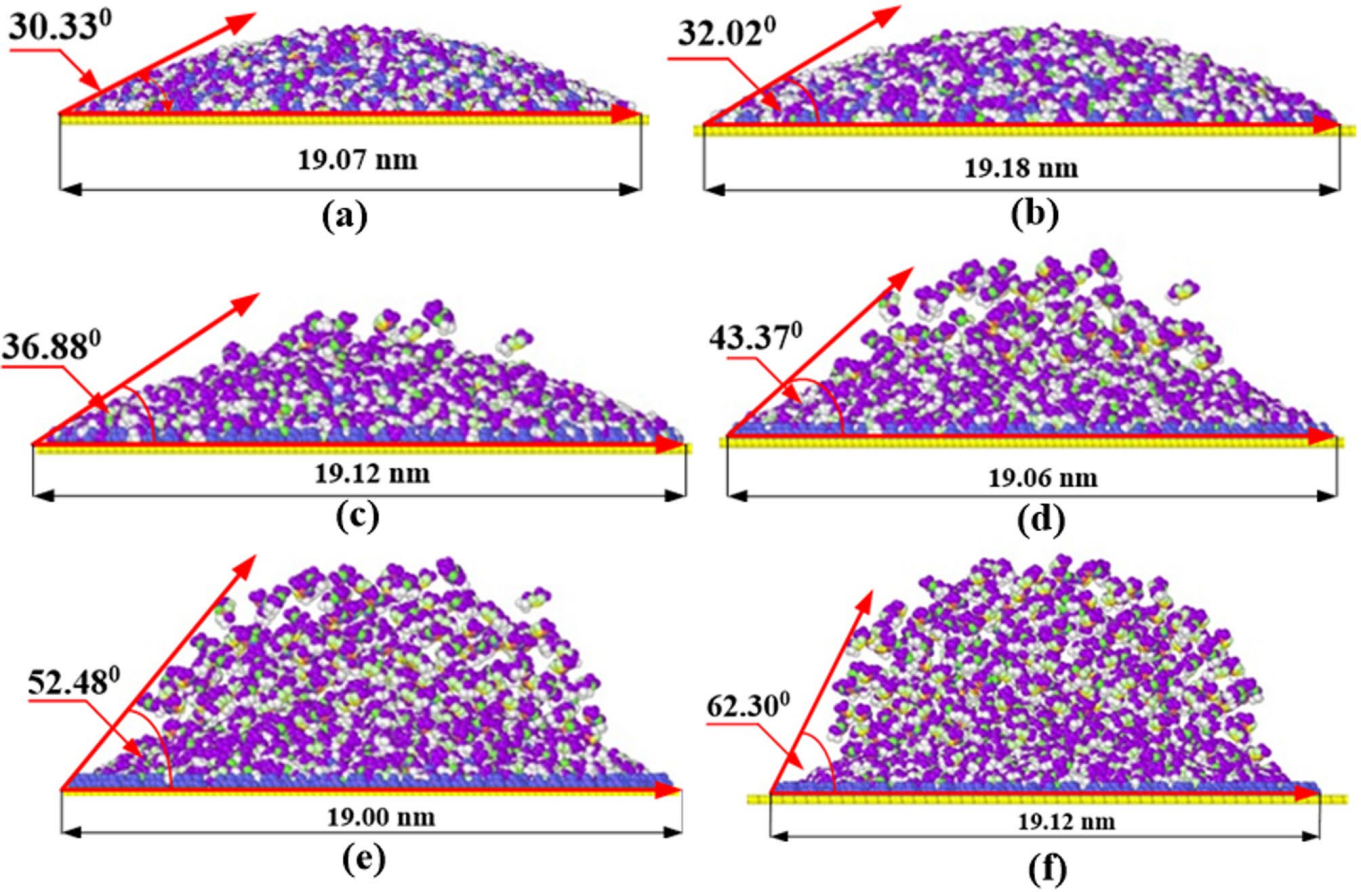
Front views of the atomic positions of electrolyte molecules at the edge of a graphene surface after 20 picoseconds of interaction, under varying surface charges: (**a**) 0.00, (**b**) 0.03, (**c**) 0.06, (**d**) 0.09, (**e**) 0.12, and (**f**) 0.15.

Figure 5 presents front views of the atomic positions of electrolyte molecules at the edge of a graphene surface after 20 picoseconds of interaction under varying surface charges. The contact angle was measured using ImageJ software. In this method, a snapshot of the EMIMBF₄ electrolyte on the graphene surface was analyzed using the tangent method, where a tangent is drawn at the contact point between the solid and liquid surfaces, and the angle is calculated using the software’s analysis tools. Several studies have reported that the geometrical contact angle measured at small scales may not always correspond directly to a macroscopic physical property such as surface tension or wettability^31^. However, in our study, the contact angle is mainly used as a relative comparison between different surface charge conditions. Therefore, although there may be minor uncertainties in the absolute value of the contact angle, these do not affect the overall trends or conclusions of our work.

The panels show the droplet profile, and the contact angle (in degrees) evaluated for surface charge ranging from 0.00 to 0.15 eV. At a low surface charge, such as 0.00 eV, the contact angle measured is 30.33°, indicating high wettability as the droplet spreads widely on the graphene surface. As the surface charge increases, the contact angle also increases. For example, at approximately 0.06 eV, the contact angle rises to 36.88°, signifying a significant decrease in wettability. At 0.15 eV, the contact angle stabilizes at 62.30°, showing that the electrolyte’s ability to interact with the graphene surface decreases with higher charge. The results derived from these measurements suggest that there is a decrease in spreading of the droplet on the graphene surface at higher surface charge densities; the droplet has more rounded shape with greater contact angle. This trend emphasizes the abilities of surface charge modulation to create wettability in cases such as applicability of electro wetting and droplet control on conducting thin films in which accurate regulation of electrolyte action is required.

**Fig. 6:**
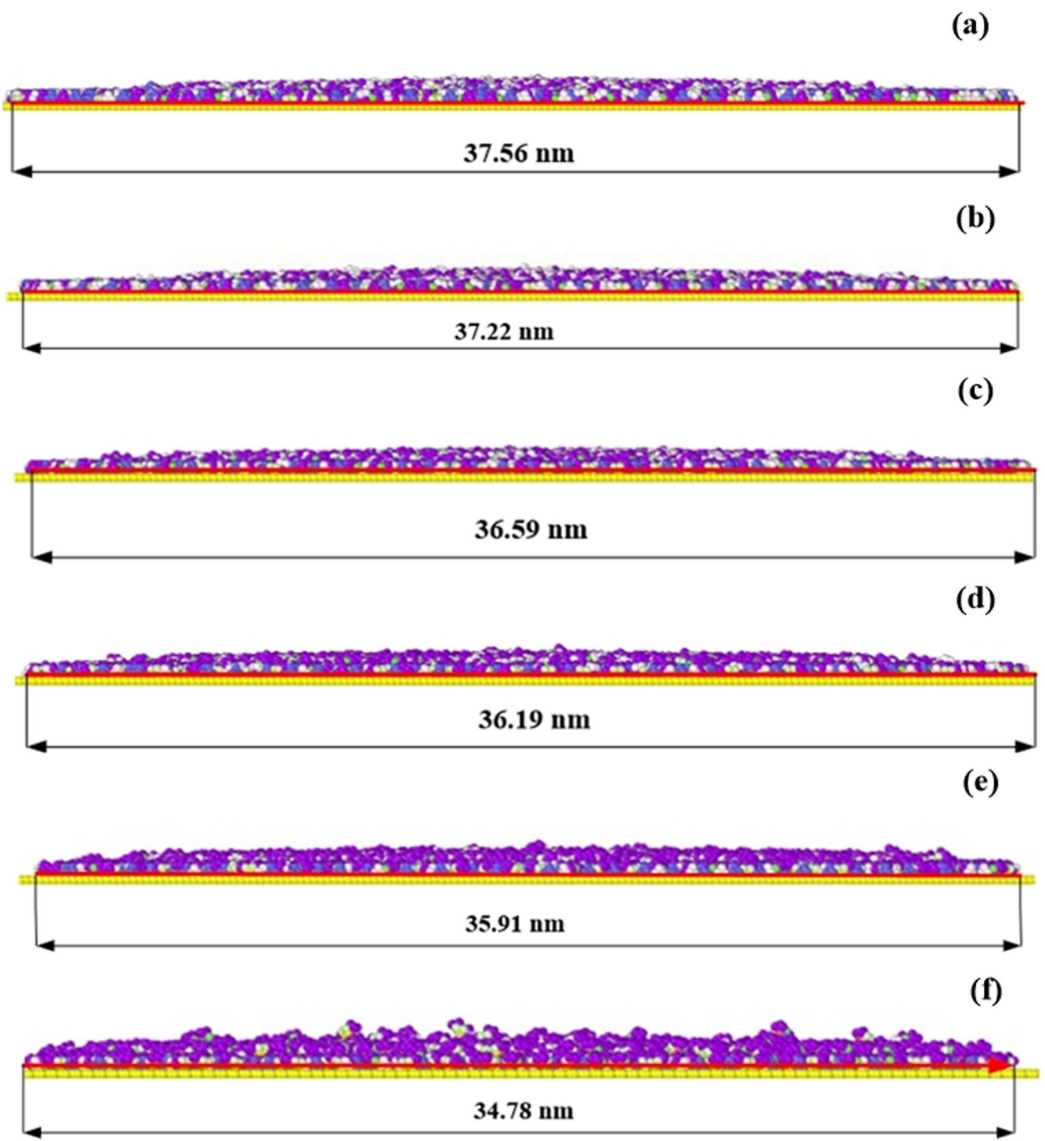
Front views of the atomic positions of electrolyte molecules at the edge of a graphene surface after 100 picoseconds of interaction, under varying surface charges: (**a**) 0.00, (**b**) 0.03, (**c**) 0.06, (**d**) 0.09, (**e**) 0.12, and (**f**) 0.15.

Figure 6 presents front views of the atomic positions of electrolyte molecules at the edge of a graphene surface after 100 picoseconds of interaction under varying surface charges. The panels (a) through (f) illustrate a step increase in the surface charge density at the graphene surface, from 0.00 to 0.15 eV on a step of 0.03 eV. These results indicate that when the surface charge at the graphene surface is increased, the charged surface attracts the electrolyte molecules, and the spread of the latter becomes more compact. The lateral spread widths are as follows: (a) 0.00: 37.56 nm, (b) 0.03: 37.22 nm, (c) 0.06: 36.59 nm, (d) 0.09: 36.19 nm, (e) 0.02: 35.91 nm, (f) 0.15: 34.78 nm, these results suggest that increasing the surface charge on graphene enhances the attraction between the charged surface and the electrolyte molecules, causing the electrolyte to become more localized and compact. This leads to the formation of a compressed layer where the lateral distribution of the electrolyte decreases with an increase in the surface charge density of the substrate wall.

These results present important implications for high charge control governing wettability and electrolyte architecture on conductive systems. By changing the surface charge, it is possible to control the spatial localization and density of the electrolyte layers that is important for the construction of new functional materials and devices in such areas as energy storage, electro wetting, nanofluidic and others.

### Influence of charge variation on wettability

**Fig. 7:**
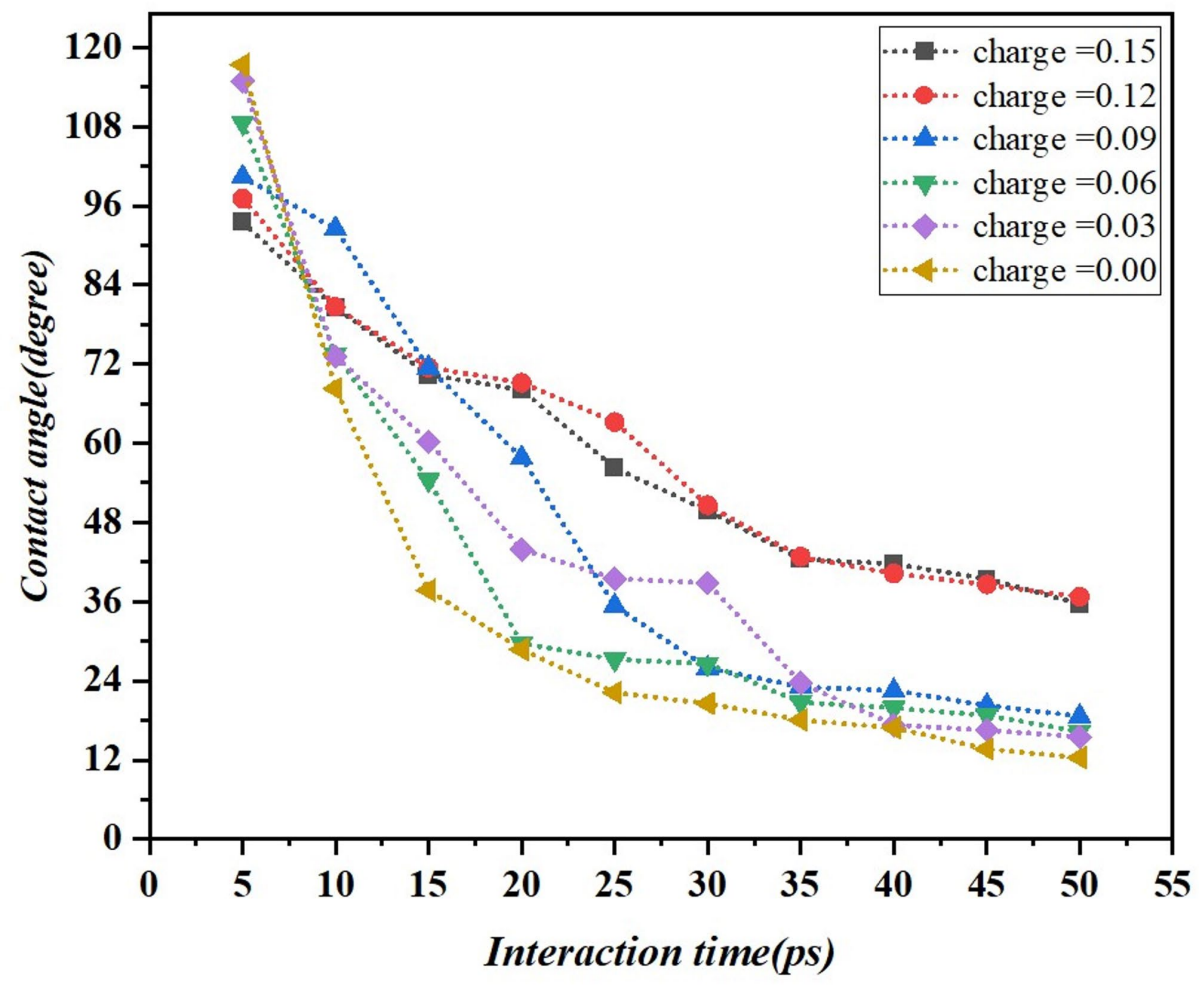
Variation of contact angles over time under different surface charge conditions.

Figure 7 illustrates the effect of interaction time on the contact angle of a droplet, as determined through molecular dynamics simulations under varying charge conditions. The results clearly demonstrate that wetting improves with an increase in interaction time, a trend that is consistent across all charge levels. Importantly, charge values charged at higher values at the onset in contact angle measurements, which denote a surface exhibiting greater hydrophobicity^9,32^. For example, at a charge of 0.15 eV, the contact angle is above 100° and decreases gradually but remains relatively high all the same. Consequently, charges 0.03 eV and 0.00 eV present lower initial CA values and rapidly achieve lower constant values, indicating higher wettability^33,34^. That is, after about 30 to 40 ps for each curve, the value indicates an approximate contact angle that is characteristic of equilibrium. This trend underlines the fact that the change in the charge can influence wettability, with the increase of the charge reducing droplet spreading and lowering the charge that causes increased spreading and therefore, fundamentals to design surfaces with hydrophobic or hydrophilic characteristics are met. Graphene’s high surface energy and π-electron interactions with the ionic EMIMBF_4_ enable better electrolyte spreading, ensuring efficient ion transport and reduced interfacial resistance. In our MD simulations, the interaction between graphene’s π-electron system and the ionic liquid EMIMBF₄ is represented by a Combination of Vander Waals (Lennard-Jones) and Coulombic interactions. Although the π-electron cloud itself is not directly modeled (like in quantum-level simulations), its influence is accurately represented by well-parameterized force fields that represent the non-bonded interactions between graphene carbon atoms and the EMIMBF₄ electrolyte^35^. This improved wettability maximizes the effective surface area for charge storage, leading to higher capacitance and faster charge/discharge rates, also enhances electrochemical stability by providing uniform electrolyte coverage, supporting long-term cyclability. These factors collectively result in supercapacitors with superior energy and power densities, making graphene EMIMBF_4_ systems ideal for high-performance energy storage applications.

### Influence of wettability with respect to temperature and potential energy

**Fig. 8:**
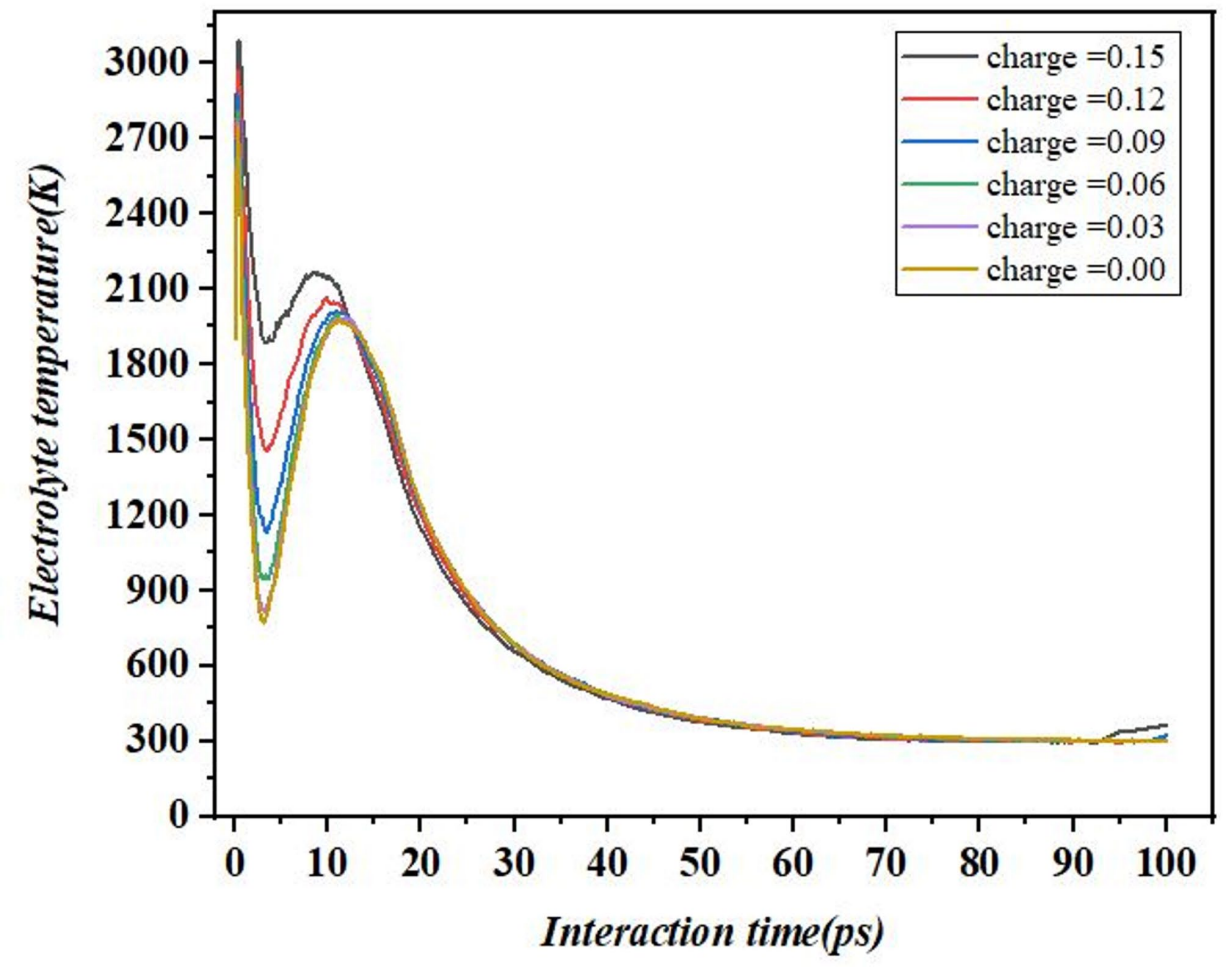
Temperature variation of individual electrolyte nanodroplets with surface charges.

Figure 8 shows the impact of the charge conditions, as simulated in molecular dynamics, on the electrolyte temperature against the interaction time. The data also show that when charging begins, the temperature rises rapidly for all values of the charge and is greater than 2000 K during the first five picoseconds. This sharp increase is probably because of the straight transfer of energy caused by the first contacts in the electrolyte. After, the temperature goes to its highest turning point, last, higher charges, for instance, 0.15 eV, elicits a steeper temperature trend around 2800 K translating into higher electrostatic interactions at higher charges. After that a cooling phase where the temperature gradually starts to reduce, this is the case because after the heating phase, the temperature just falls gradually in the cooling phase. Subsequently all charge levels come to have similar relaxation time of the order of 50 ps and settle to 100–200 K which represents different charges nearing thermal equilibrium. These results also underline the charge behavior of the initial temperature tendency where the increase of the charge results in the intensified thermal response of the electrolyte^36^. These considerations are critical for precise thermal management as evidenced by batteries applications.

**Fig. 9:**
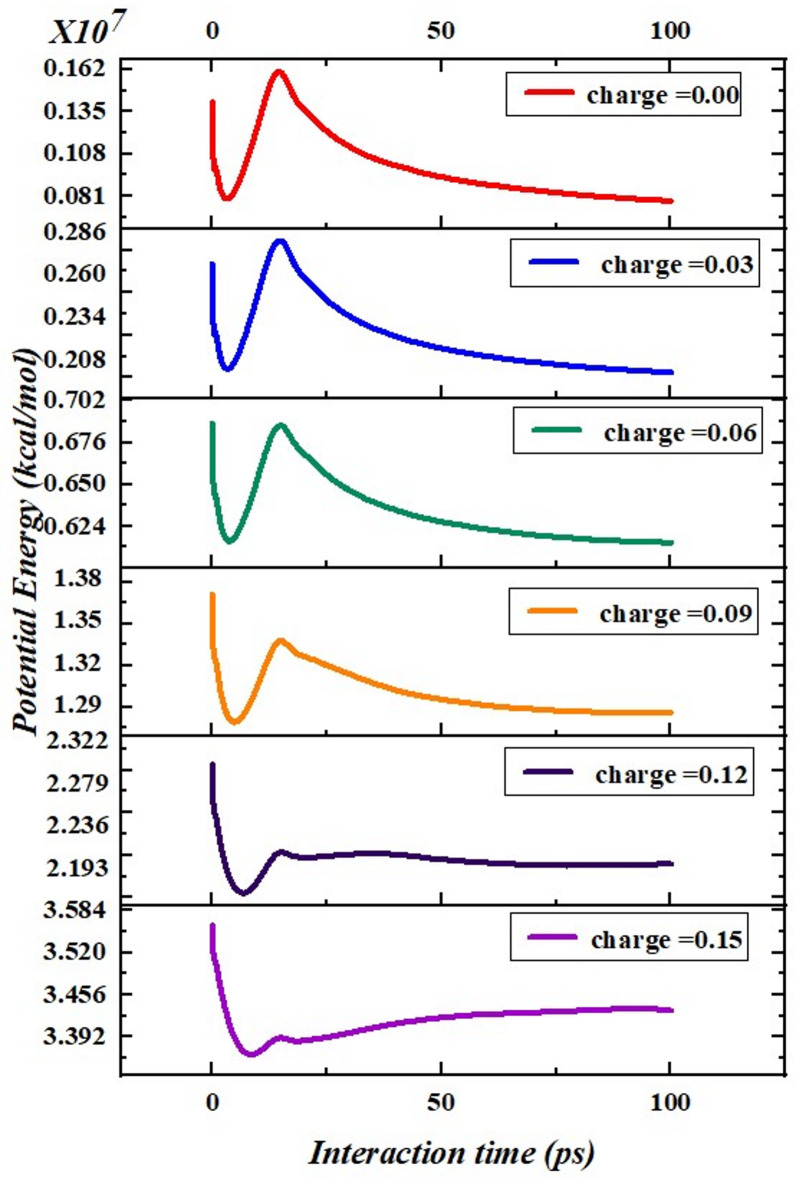
Fluctuations in potential energy with different surface charges.

Figure 9 shows the ability of EMIMBF_4_ ions in an electrolyte to interact with a graphene sheet, as modeled by a 100 ps molecular dynamics (MD) simulation, where each symbol represents a different charge. Throughout the ion charge increase from 0.00 to 0.15 eV, the initial peak and the final equilibrium P.E values are increased, proving the non-physical argument that there is an increase in the extents of electrostatic interactions between the electrolyte ions and the graphene. At 0.00 eV, the potential energy becomes as stable as 0.081 $\:\times\:$ 10^7^ kcal/mol. Therefore, when charge is raised to 0.03 eV, the ideal energy level is approximately equals 0.234 $\:\times\:$ 10^7^ kcal/mol. This upward trend continues as charge increases: For 0.06 eV, equilibrium potential energy is about 0.650 $\:\times\:$ 10^7^ kcal/mol in case of 0.09, it becomes 1.32 $\:\times\:$ 10^7^ kcal/mol at 0.12 eV, it becomes 2.279 $\:\times\:$10^7^ kcal/mol and at 0.15 eV, the level goes on to reach the highest level of 3.520 $\:\times\:$ 10^7^ kcal/mol. These results reveal a consistent pattern: As the value of charge on the electrolyte ions increases, their bond with the graphene increases proportionally. As the charges increase, the bait force becomes stronger, and it increases the potential energy needed to keep the ions from getting discharged on the graphene layer. At the beginning of the simulation, the ions are placed in a high energy configuration since they are organized close to the graphene. Its components institute a condition for equilibrium, the ions move into positions that are of least energy as the energy become balanced or equal^37–39^. This correlation between ion charge and interaction energy is useful in synthesis of energy storage materials including batteries and super capacitors. In general, enhanced electrostatic interactions can affect ion transport and adsorption and attachment features and performance of such devices. Researchers believe that the strength with which they can alter the mass of the electrolyte ions with graphene might help in enhancing other vital aspects of the energy devices, including ion conductivity and storage capability.

Electrolyte temperature significantly influences wettability and the performance of energy storage systems. As temperature increases, electrolyte viscosity decreases, promoting better spreading on the electrode surface and improving wettability. This enhances ion mobility, facilitating faster diffusion and more efficient charge/discharge cycles. For supercapacitors, higher temperatures can improve capacitance, power density, and reduce internal resistance.

**Fig. 10:**
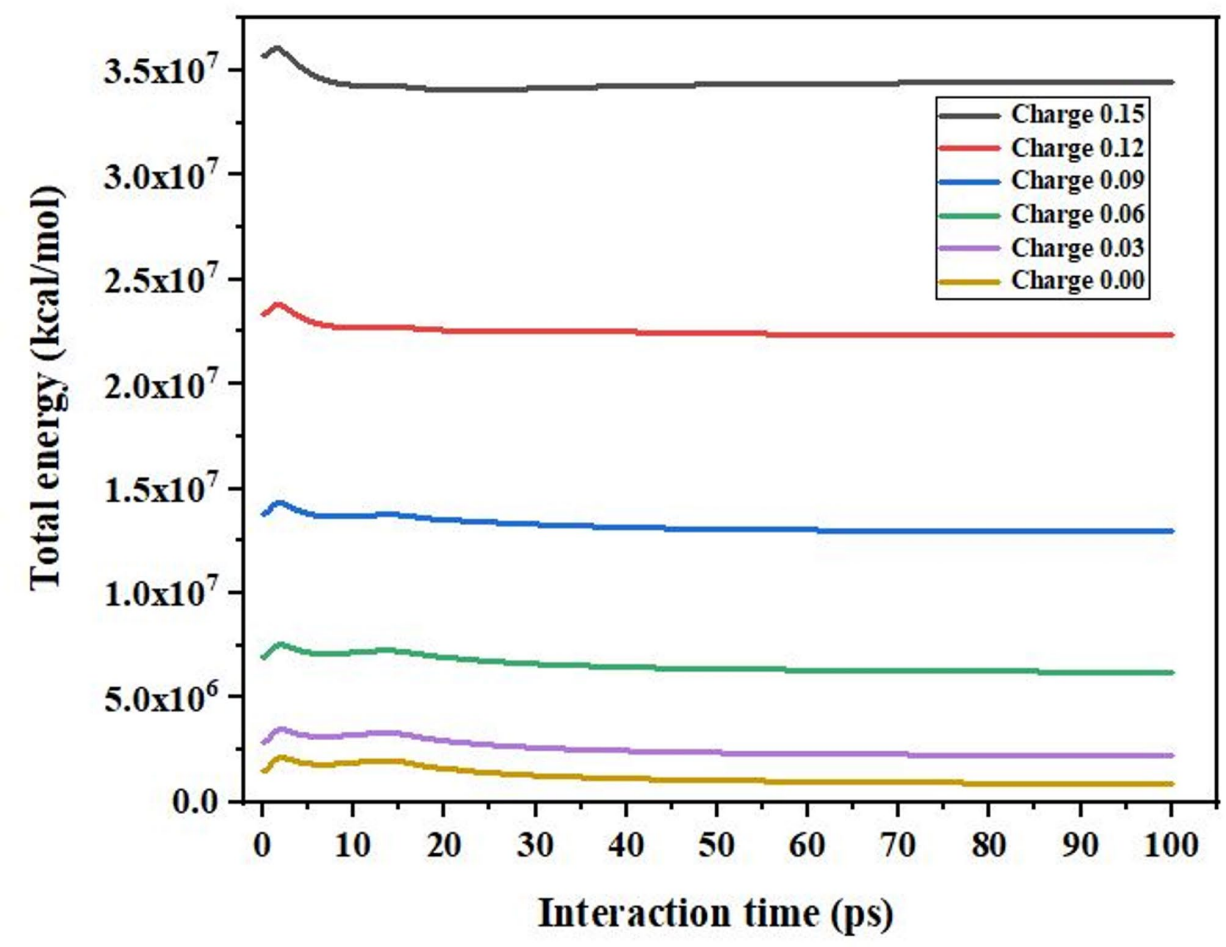
Fluctuations in total energy with varying surface charges.

In this study, the total energy is also investigated. Figure 10 illustrates how the total energy varies with interaction time for different charge values in a molecular dynamic’s simulation. The plot provides insights into the energy changes as the system evolves over time, and the importance of the influence of charge on the overall energy dynamics. First, the total energy changes due to the system performance where it comes to a stable stage later. This means that when charges are higher there is higher total energy because of enhanced electrostatic interaction between charges. Lower charges continue to stabilize the system quicker and record fewer oscillations. The first variations are sensitive to initial conditions and correspond to 0–10 ps; the flat phase corresponds to the state of equilibrium. Higher charges cause enhanced disturbances, which decelerate stabilization processes.

### **Analysis of electric force variation across different surface charges**

**Fig. 11:**
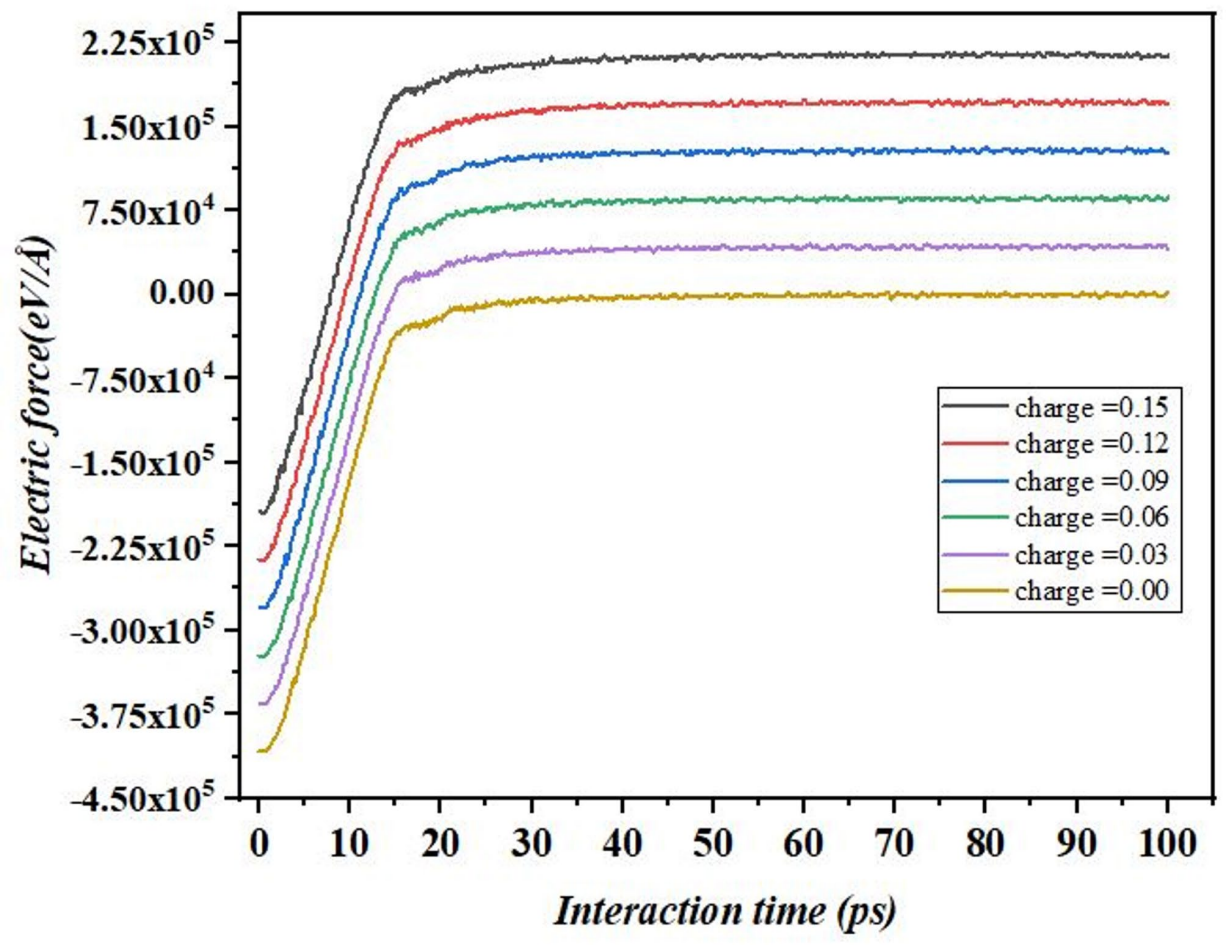
Dynamics of the electric force acting upon an assumed plane on the surface of graphene under different conditions over time.

Figure 11 demonstrates the time interaction function of electric force in molecular dynamics simulation for different atomic charges. This specification of the electric force in eV/Å is here in displayed over 100 ps for six different charge values as shown below: first 10 ps, in charge levels all the electric force increases rapidly showing an atoms response or adaptation to the electric field applied. This resistance is charging dependent and especially higher charges offer larger forces than smaller charges for instance, 0.15 eV. Following this initial phase the electric force value settles down and does not oscillate as the charge for each level of charge increases. Ions with greater charges achieve greater steady state electric force magnitudes showing thus, directly proportional, concept with atomic charge idiomatically. For instance, the force at charge 0.15 eV, is bigger than force at 0.03 eV. In contrast, at a charge of 0.00 the force remains close to zero as is expected at no electric interactions. The results obtained herein validate Coulomb’s law which asserts that electric force is directly proportional to the atomic charge under an external field^40^. This behavior is essential for anyone who is trying to comprehend the nature of electrostatic interactions in charged structures, and the behavior of electrolytes in electrochemical systems where charge distribution plays a very important role in system response.

### Density distributions for electrolyte molecules

**Fig. 12:**
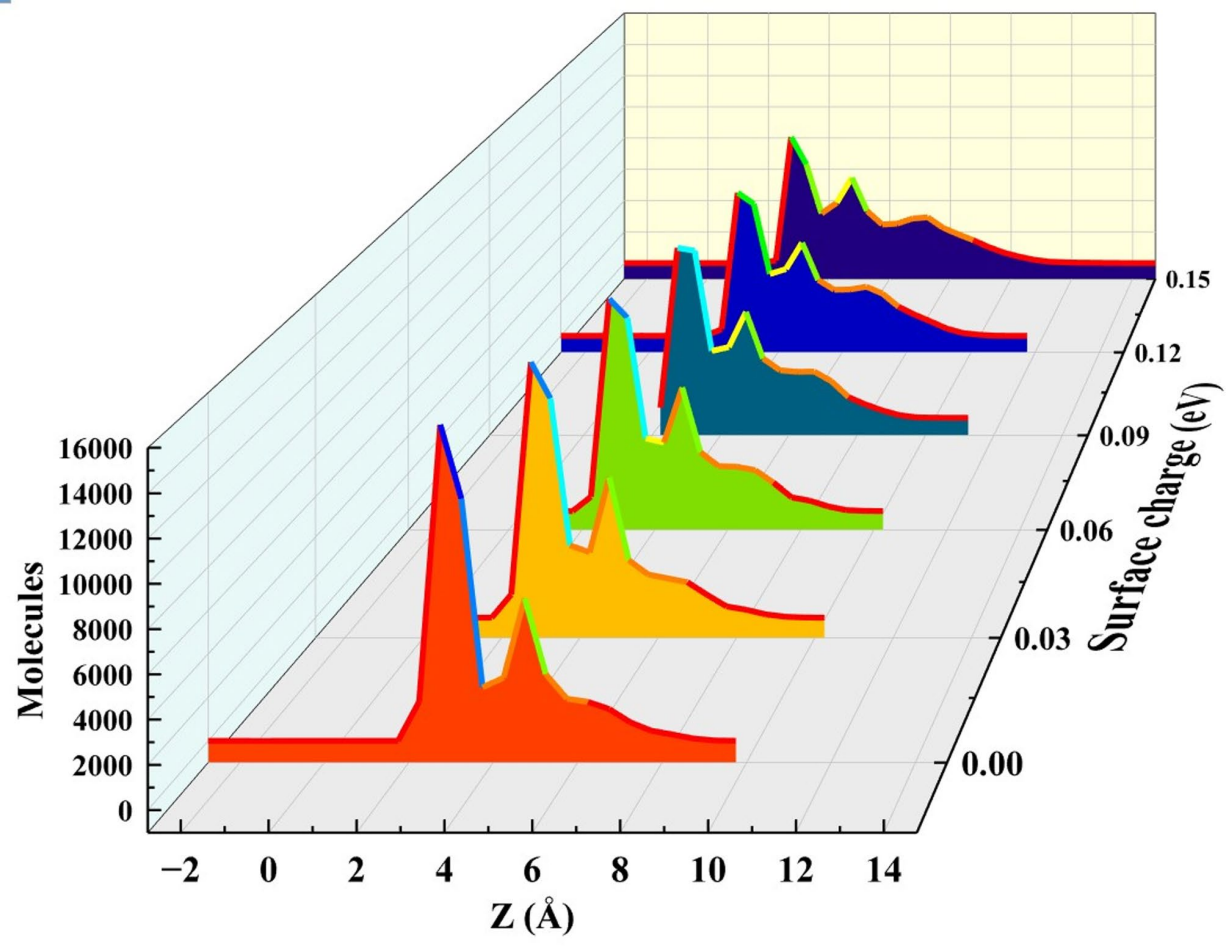
Distribution of electrolyte screening length of the molecular profile on the graphene surface under different surface charge states.

The Fig. 12 illustrates the density distributions of EMIMBF_4_ electrolyte molecules on a graphene surface as a function of distance (Z, in Å) under varying surface charge (0.00 to 0.15 eV). The observed decrease in the height of the first peak (closest to the surface) with increasing surface charge can be attributed to several factors. Firstly, enhanced electrostatic repulsion plays a key role: a positively charged surface repels EMIM⁺ Cations, while a negatively charged surface repels BF4⁻ anions, reducing their density near the surface. Secondly, molecules may reorient to minimize unfavorable interactions, such as tilting of EMIM⁺, leading to less dense packing in the first layer. Also increased surface charge attracts counterions for screening, causing a more diffuse layering and lowering the first peak’s intensity. Lastly, steric crowding decreases as electrostatic forces dominate over Vander Waals interactions at higher charges, further reducing the density of the first layer. This indicates that electrostatic effects and molecular adjustments are critical in determining the electrolyte’s structuring near charged surfaces^38,41–43^.

As the Z-distance is increased, the concentration of molecules decreases to suggest that the electrostatic effects of the graphene surface are minimal as the distance from the surface increases. This charge-dependent layering of electrolyte molecules near the graphene surface is important in deciphering electrochemical behaviors in systems such as super capacitors and batteries where surface charges play a major role in structuring the electrolyte and the diffusion of ions^45–48^. This observation of graphene charges plays a crucial role in controlling the distribution of electrolyte, which, in turn, affects electrochemical performance.

### Mean square displacement (MSD) and diffusion coefficient

**Fig. 13:**
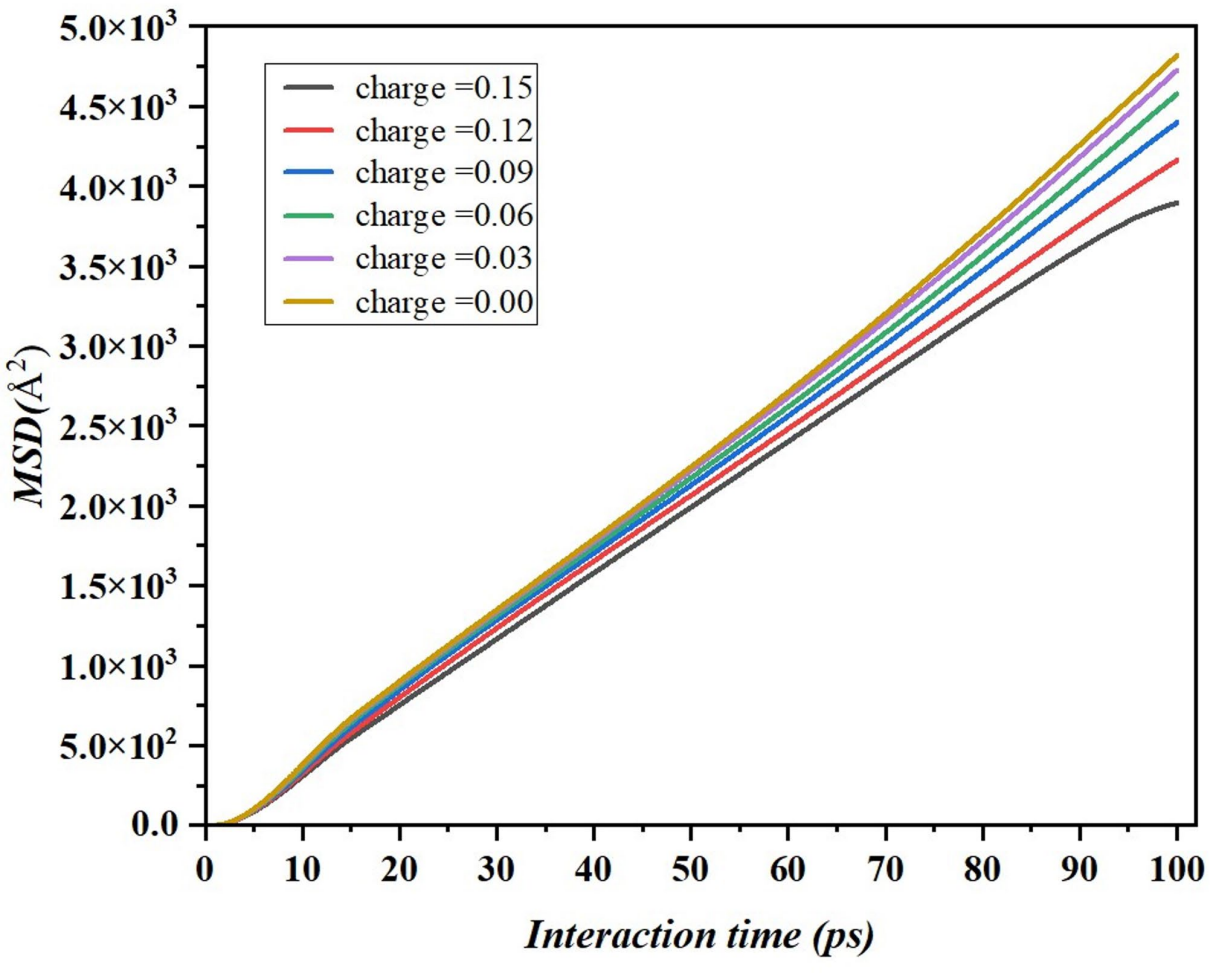
MSD plots of electrolyte molecules on the graphene surface in different surface charge environments.

The positions and motions of the electrolyte atoms are described normally as functions of boundary nodes with certain changes. Consequently, the electrolyte molecules come across each other, and the molecules on the graphene surface may switch to a different route at each time step^48^. It is rather difficult to monitor the movements of electrolyte molecules in real time, because each molecule’s path along the time is rather random and rather uneven^49^. The displacement travelled by molecules can be calculated with the help of MSD of electrolyte molecules. The MSD of a molecule is obtained based on current position with respect to time^50,51^. From the work of Bian et al.^52^, Eq. 5 can be interpreted as the average MSD for many electrolyte molecules available.

5 $$\:\stackrel{-}{MSD}\left(t\right)=\:\sum\:_{j=1}^{N}\frac{{\left\{{r}_{i}\left(t\right)-{r}_{i}\left(0\right)\right\}}^{2}}{N}$$

where N is the total number of atomic sites to understand the diffusion behaviors, the calculated MSD has been plotted against the interaction time which is shown in Fig. 13. The results reveal that MSD increases with interaction time, and shows linear relationship^53^, which means that the particles continue to diffuse in all charges^43,54^. But just as it was stated earlier, the rate of increase in MSD varies with charge. The highest MSD value was recorded for particles with 0.00 eV charge (yellow line) throughout the interaction time, while the lowest MSD value corresponds to particles with 0.15 eV charge (black line). This sort of trend indicates that as the charges increase, the new particle displacement is likely to be decreased because of resistors such as electrostatic charges or other similar forces that tend to hinder the movement of the particles. On the other hand, the lower charges lead to higher MSD as demonstrated by the data collected Average Displacement Amplitude (ADA). These findings relate well with previous results of diffusion coefficients decreasing with increasing charge and thus provide evidence towards the hypothesis that the charge of particles drives diffusion in the system.

**Fig. 14:**
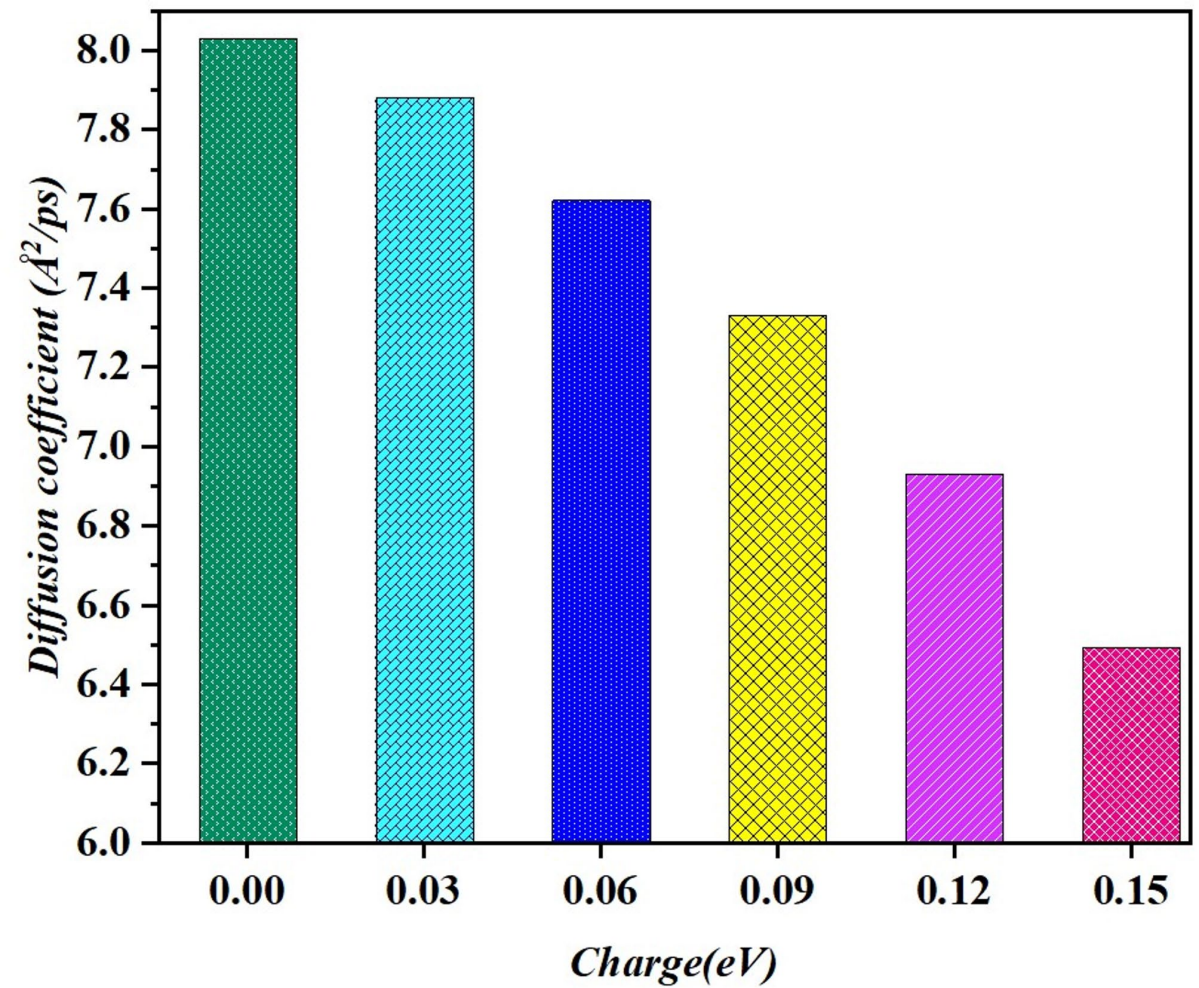
Diffusion coefficient of electrolyte nanodroplets on the graphene surface under different surface charge states.

MSD, where the molecules’ dispersal within the system under study is depicted, is applied to assess dynamic properties of a confined nanofluidic system; its differentiation is often used for this purpose. More specifically, this paper compares the effect of various surface charges on the diffusion dynamics of electrolytes upon the graphene surface. The diffusion coefficient (D) is described as the ratio of the mean square displacement (MSD) slope (m) to 6, where the units of ‘m’ are Å^2^/ps^55^. Figure 14 illustrates an analysis of the diffusion coefficient and charge in the molecular dynamic simulation of particles to show how mobility of particles is influenced by charge fluctuations. On the x-axis there is the voltage applied to the particles, in electron volts, eV, and the y-axis is the diffusion coefficient, which describes the rate at which the particles move in the system. The results show also that there is a strong negative correlation between the degree of charge and the diffusion coefficient. Thus, the integrated charge of 0.00 eV gives the highest value of diffusion coefficient ≈ 8.0 Å^2^/ps suggesting high particle mobility. As the charge increases further, a minor reduction in diffusivity is observed, this being since as the charge increases with the increment of 0.03 eV the diffusion coefficient decreases.

This trend is also observed for further increases in charge, while the diffusion coefficient gradually decreases, in the range of 6.0 Å^2^/ps for a charge of about 0.15 eV. These results imply that higher charge negatively affects the system’s particle diffusion and mobility since charge causes electrostatic forces or increases the particle’s resistance when moving within the system. Such observations suggest ways to control charge levels to achieve the desired diffusivity, particularly for material systems where the particle transport properties are important, as in the case of electrolytes in batteries. This idea could be useful in optimizing the electrolyte’s performance of balancing charges in order to attain specific diffusion rates.

### Analysis of resistance force (F_R_)

The interaction force between a graphene surface and the ionic liquid electrolyte 1-ethyl-3-methylimidazolium tetrafluoroborate (EMIMBF_4_) is influenced by the surface charge on the graphene. When the surface charge increases from 0 to 0.15 eV, the Resistance force experienced by the electrolyte decreases. This phenomenon can be explained by the electrostatic interactions between the charged graphene surface and the ions in EMIMBF_4_, which consists of EMIM⁺ (Cation) and BF4^−^ (Anion).

**Fig. 15:**
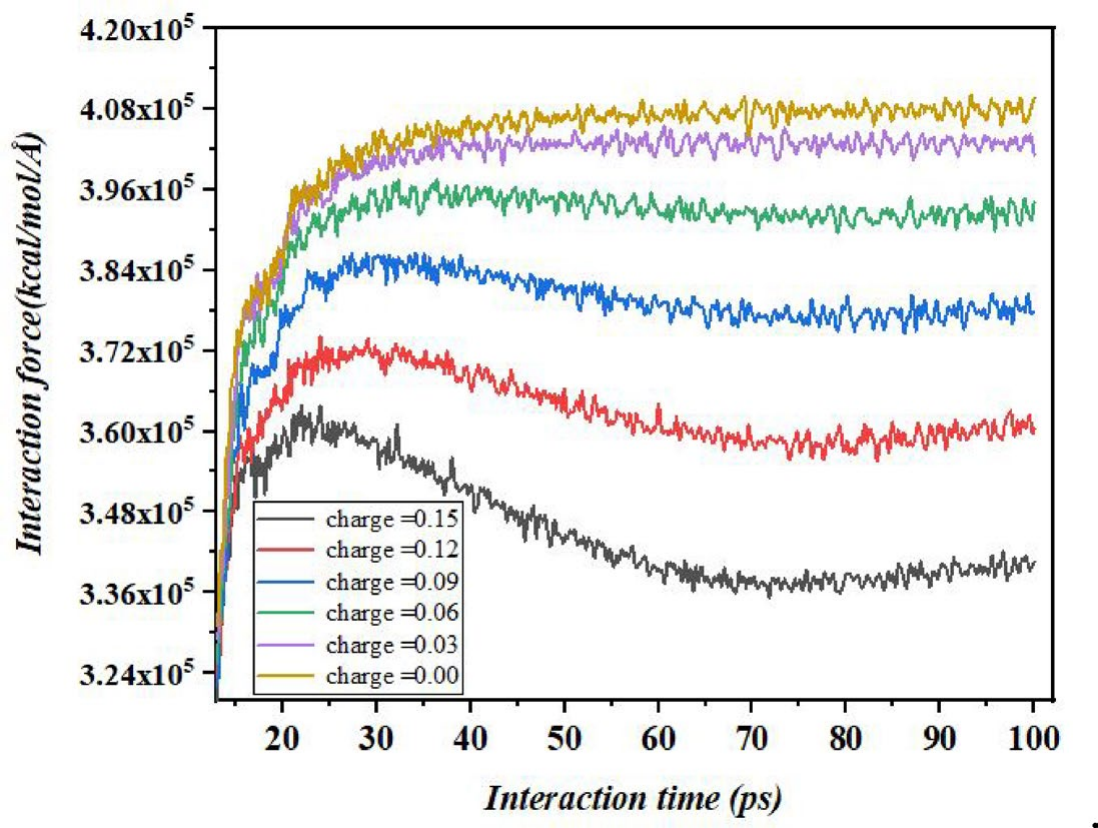
Resistance force on an assumed plane surface over time under different conditions on a graphene surface.

As the graphene surface acquires a positive charge, it repels the positively charged EMIM^+^ ions and attracts the negatively charged BF4⁻ ions due to Coulombic forces. Similarly, a negatively charged surface would repel BF4^−^ and attract EMIM⁺. This charge-dependent interaction disrupts the structured layering of the ionic liquid near the graphene surface, reducing the steric and electrostatic Resistance to ion movement. The increased surface charge enhances same-charge repulsion and opposite-charge attraction, weakening the cohesive interactions within the electrolyte’s double layer. Consequently, the energy barrier for ion displacement decreases, leading to a reduction in the Resistance force^56,57^. This behavior is consistent with molecular dynamics studies of ionic liquids on charged surfaces, where higher surface charge reduces the viscosity and friction of the electrolyte by altering ion orientation and distribution. As shown in the Fig. 15, the interaction force increases sharply during the initial phase of the simulation (up to approximately 20–30 ps), indicating the rapid establishment of interfacial interactions between the electrolyte nanodroplet and the charged surface^36,41,58^. For instance, at a surface charge of 0.00 eV the system exhibits the highest interaction force (4.1 $\:\times\:$ 10^5^ kcal/mol/Å), whereas at 0.15 eV the interaction force is significantly lower (3.3 $\:\times\:$ 10^5^ kcal/mol/Å) and shows a slight decay after the initial peak.

## Conclusion

The molecular dynamics simulations provide a comprehensive analysis of electrolyte-graphene interactions, emphasizing key parameters influencing droplet behavior, wettability, and electrostatic interactions. Key findings include the equilibrium conformation of the electrolyte droplet on a 40 nm $\:\times\:$ 40 nm graphene surface, where the droplet stabilizes at around 100 ps, showing a circular shape with a diameter of 36.26 nm, and a homogeneous electrolyte layer. This indicates stable interactions between the electrolyte and graphene. Surface charge plays a significant role in altering wettability, with the contact angle increasing from 30.33° at 0.00 eV, to 66.88° at 0.15 eV. This demonstrates reduced wettability as charge increases, offering a method to control droplet spreading, which is useful for applications such as electro wetting. Furthermore, the lateral spread width of the droplet decreases as surface charge density increases, with values of 37.56 nm at 0.0 eV, and 34.78 nm at 0.15 eV, highlights the compact distribution of the electrolyte at higher charges.

Interaction time also influences wettability, with longer times leading to reduced contact angles, especially at higher charges. For example, at 0.15 eV, the contact angle decreases from an initial value of over 100° over time, reflecting evolving interactions. Temperature simulations show a rapid initial increase in temperature for higher charge densities, peaking at 2800 K and cooling to 100–200 K, indicating the thermal effects of charge density. Additionally, potential energy increases significantly with surface charge density, from 0.081 $\:\times\:$ 10^7^ kcal/mol to 3.520 $\:\times\:$ 10^7^ kcal/mol, suggesting stronger electrostatic interactions. Electric force values also follow a similar trend, with higher charges leading to greater forces, supporting Coulomb’s law. Electrolyte molecule density increases near the graphene surface with higher charge densities, indicating enhanced layering effects. Finally, MSD analysis shows that higher surface charges decrease diffusion rates, emphasizing the role of charge in controlling particle mobility, critical for optimizing electrolyte performance in energy storage systems. These findings provide insights into electrolyte behavior for advanced energy and nanofluidic applications.

## Data Availability

The data used and/or analyzed during the current study are available from the corresponding author upon reasonable request.
